# The Impact of Individual Differences, Types of Model and Social Settings on Block Building Performance among Chinese Preschoolers

**DOI:** 10.3389/fpsyg.2018.00027

**Published:** 2018-01-30

**Authors:** Mi Tian, Zhu Deng, Zhaokun Meng, Rui Li, Zhiyi Zhang, Wenhui Qi, Rui Wang, Tingting Yin, Menghui Ji

**Affiliations:** ^1^Department of Psychology, The Chinese University of Hong Kong, Hong Kong, China; ^2^School of Psychology, Nanjing Normal University, Nanjing, China; ^3^School of Art and Literature, Shihezi University, Xinjiang, China; ^4^School of Foreign Languages, Huazhong University of Science and Technology, Wuhan, China; ^5^School of Foreign Languages and Cultures, Nanjing Normal University, Nanjing, China; ^6^School of Foreign Studies, Nanjing Forestry University, Nanjing, China; ^7^Nanjing Liuyi Kindergarten, Nanjing, China

**Keywords:** block building, individual differences, types of model, social settings, Chinese preschoolers

## Abstract

Children’s block building performances are used as indicators of other abilities in multiple domains. In the current study, we examined individual differences, types of model and social settings as influences on children’s block building performance. Chinese preschoolers (*N* = 180) participated in a block building activity in a natural setting, and performance was assessed with multiple measures in order to identify a range of specific skills. Using scores generated across these measures, three dependent variables were analyzed: block building skills, structural balance and structural features. An overall MANOVA showed that there were significant main effects of gender and grade level across most measures. Types of model showed no significant effect in children’s block building. There was a significant main effect of social settings on structural features, with the best performance in the 5-member group, followed by individual and then the 10-member block building. These findings suggest that boys performed better than girls in block building activity. Block building performance increased significantly from 1st to 2nd year of preschool, but not from second to third. The preschoolers created more representational constructions when presented with a model made of wooden rather than with a picture. There was partial evidence that children performed better when working with peers in a small group than when working alone or working in a large group. It is suggested that future study should examine other modalities rather than the visual one, diversify the samples and adopt a longitudinal investigation.

## Introduction

Children’s block building has been investigated for over a century (Froebel, 1895), and its relevance is documented in recent studies (Casey et al., 2012; Ramani et al., 2014; Newman et al., 2016). In preschool settings, children are provided with wooden unit blocks of varying shapes and sizes for the purposes of free play; children are also sometimes asked to copy a model or a picture, with more difficult tasks requiring symbolic representation (Otsuka and Jay, 2016). Such building activity is, more often than not, recognized as an effective way to promote children’s overall development (Rogers, 1985), literacy skills (Isbell and Raines, 1991; Wellhousen and Giles, 2005; Cohen and Uhry, 2011), social skills (Cohen and Uhry, 2007), mathematic skills (Casey et al., 2012) and spatial skills (Ramani et al., 2014; Cohen and Emmons, 2017).

One area of interest has been block building as an indicator of the development of symbolic representation, which involves a complex process associated with problem-solving, calculation and abstract thinking abilities (Diana and Test, 2011; Uhry and Cohen, 2011; Otsuka and Jay, 2016). There are several gaps in the literature. One aspect of block building that has not been studied concerns preschoolers’ ability to copy models, either a wooden model or a picture. An understanding of preschoolers’ block building skills under these two conditions might help shed some light on their psychological and cognitive development. More research is also needed on individual differences in block building skills based on gender (e.g., Goodson, 1982; Saracho and Spodek, 1995; Hanline et al., 2001; Casey et al., 2008; Kersh et al., 2008) and year in preschool (e.g., Stiles-Davis, 1988; Stiles and Stern, 2001), as the results of the extant literature are far from consensus. Lastly, the role of social settings (individual or group) (e.g., Hanline et al., 2001; Casey et al., 2008) has not been fully identified.

To this end, the present study examines gender differences, grade level (K1, K2, and K3, i.e., 1st, 2nd, and 3rd year in preschool or kindergarten, e.g., Shu et al., 2008), types of model (wooden model or pictures), and social settings (working individually or in a group) as predictors of block building performance. Block building was assessed using multiple measures, assessing a wide range of skills. The following sections review the relevant research on individual differences, types of model and social settings in relation to preschoolers’ block building.

### Individual Differences

Gender differences in block building performance have been investigated since the late 1950s (e.g., Farrell, 1957; Margolin et al., 1961; Clark et al., 1969). Early work in younger children samples suggested that more boys play with blocks than girls, boys spend more time in the block area (Farrell, 1957), and girls are more interested in non-block activities compared with boys (Margolin et al., 1961). More recent studies (e.g., Caldera et al., 1999; Snow et al., 2016) have obtained inconsistent results with regard to gender differences in children’s preference for block building. Importantly for the current study, these studies focused on preference rather than process. That is, they did not compare boys and girls on block building skills in terms of spatial reasoning, for example as seen in the structural features and representational quality of children’s constructions.

There also appear to be individual differences based developmental change in block building skills, documented in research in the 1930s (e.g., Hulson, 1930; Guanella, 1934) as well as more recent research (Stiles-Davis, 1988; Stiles and Stern, 2001). Several stage models have been proposed. For example, Guanella (1934) described five stages: non-structural use of blocks in late infancy; piles or rows of blocks; bi-dimensional use of blocks; tri-dimensional use of blocks; and representational play. Johnson (1983) described seven stages: carrying blocks around; making rows or piles; bridging; enclosures; decorative patterns with symmetry; naming of block constructions; and dramatic play with block constructions.

Other research has focused on children’s block building skills in relation to their cognitive development (e.g., Goodson, 1982; Stiles-Davis, 1988). For instance, Goodson (1982) found a positive correlation among children’s skills in building arches, planning, and perception. Reifel and Greenfield (1982) also noted that integration and dimensionality in children’s block constructions are in line with the complexity of cognitive structures. However, these studies concerned only a subset of block construction skills, without a comprehensive focus on the construction’s spatial, balance and structural features.

### Types of Model

Traditional semiotics defined two components of a sign: the “signifier” and the “signified” (Saussure, 1916). “The ‘signifier’ is the physical form of the sign in words, images or sounds. The ‘signified’ is the mental concept referred to, its meaning” (Marsh and Millard, 2000, p. 78). By contrast, social semiotics “interprets language within a socio-cultural context, in which culture itself is interpreted in semiotic terms–as an information system” (Halliday, 1978, p. 2). From the social semiotics perspective, block building activities, to some extent, can be taken as the interplay between “signifier” (i.e., the symbolic representation of concrete objects or pictures in the real world) and “signified” (i.e., the realization of abstract meaning or mental concepts with wooden blocks in different shapes and sizes) in a social context. Block building becomes a special approach for preschoolers to convey abstract mental concepts by mapping symbolic representations into unit blocks they build based on their experiences.

Thus from the perspective of semiotics, preschoolers’ block building performance can be understood as a reflection of their interpretation and expression of abstract meaning. However, only a few studies (e.g., Sluss and Stremmel, 2004; Cohen and Uhry, 2011) have examined symbolic representation in block building. For example, Cohen and Uhry (2011, p. 80) asked 4-year-old preschoolers in a culturally diverse classroom “to name and describe completed block structures to consider the meaning and learning represented through play experiences.” The results showed that preschoolers built the block structures based on their personal life experiences in a social context, e.g., children described their block building based on home or school experience. In the current study, we extended this research by testing children’s block building skills when presented with two types of symbolic representation (a wooden model vs. pictures). Developmentally, it is assumed to be easier to replicate a wooden model rather than the symbolic presentation provided by pictures (e.g., Greenfield and Schneider, 1977; Beagles-Roos and Greenfield, 1979).

### Social Settings

Social settings refer to the presence or absence of peers during block building. Although children sometimes play alone with blocks, blocks often encourage group play, and children are more likely to engage in a large cluster play within the block corner than in other areas of the classroom (Kinsman and Berk, 1979). Block building in a group setting has been shown to encourage preschoolers’ prosocial behaviors such as smiling and helping, to promote communication and cooperation, and to discourage antisocial behaviors such as throwing blocks and fighting with others (Rogers, 1985). However, there may be gender differences in that boys have been found to be more influenced by the social context than girls during block building activities (Sluss and Stremmel, 2004).

Different studies have used various sizes to define “group,” such as four children (e.g., Rogers, 1985), five children (e.g., Isbell and Raines, 1991) and ten children (e.g., Hanline et al., 2001). These studies compared individual and group block building, but no further comparison was conducted on the effects of group size (Hanline et al., 2001). Besides, there were also some studies (e.g., Cohen, 2006, 2015; Cohen and Uhry, 2007) that attempted to compare different group sizes on block building performance by allowing the group members to cooperate with each other during block building. Berk (1976) identified five levels of group size in children’s play: individual, 2-member group, 3-to-5 member group, 6-or-more member group and total class group. Kinsman and Berk (1979) classified four levels of social settings in children’s block building activity, i.e., individual block building, 2-member group, small cluster and large cluster. It should be noted that, the peer cooperation in groups might confound the effect of social settings itself, since members playing in a group environment might either work together or alone. For those working together in groups, it would be rather difficult to tell whether the social settings or peer cooperation contribute to children’s block building performance. In this respect, we observed the classification of earlier studies (e.g., Berk, 1976; Kinsman and Berk, 1979) by examining three levels of social settings (i.e., individual block building, building in a 5-member group and building in a 10-member group), but each of them worked alone without peer cooperation.

### The Present Study

The present study examines the impact of gender and school level as individual differences, types of model (wooden model or picture) and social settings (individual, in a 5-member group, in a 10-member group) on block building performance among Chinese preschoolers. Multiple established measures were used to assess the full range of block building skills. There were three research questions:

(1) Will block building performance vary by gender and year among Chinese preschoolers?
(2) When asked to replicate a model, will block building performance vary depending on whether the model is a wooden model or a picture?
(3) Will block building performance vary depending on whether children work alone, in a group of five children, or a group of 10 children?

## Materials and Methods

### Participants

A total of 180 preschoolers from a public kindergarten in Nanjing city in Jiangsu province in China, ranging from K1 to K3 volunteered to participate in the block building study. Participants were from middle class families in order to keep family socioeconomic status (SES) homogeneous. Signed consent forms were obtained from parents. The experiment was approved by the ethics committee of Nanjing Normal University. Participants’ detailed demographic information is reported in **Table 1** below.

**Table 1:** Summary of participants’ demographic information (*N* = 180).

Grade level	Age range (years)	Group sizes				
		*M*	*SD*	Boys	Girls	Total
K1	3.33–4.26	3.78	0.25	29	31	60
K2	4.33–5.96	4.93	0.31	31	29	60
K3	5.30–6.24	5.73	0.25	30	30	60

### Materials

#### Blocks

At least 2000 unite blocks available to them, with 23 different shapes and sizes, including short board, medium plate, long board, small semicircle, semicircle, small curved surface, big circle, small triangles, triangles, sector, semi arches, small arch, small cubes, small rectangle, Gothic gate, small square column, square column, thin cylinder, small cylinder, middle cylinder, big cylinder, small curve A, and small curve B.

#### Wooden Model of the Tower

**FIGURE 1:**
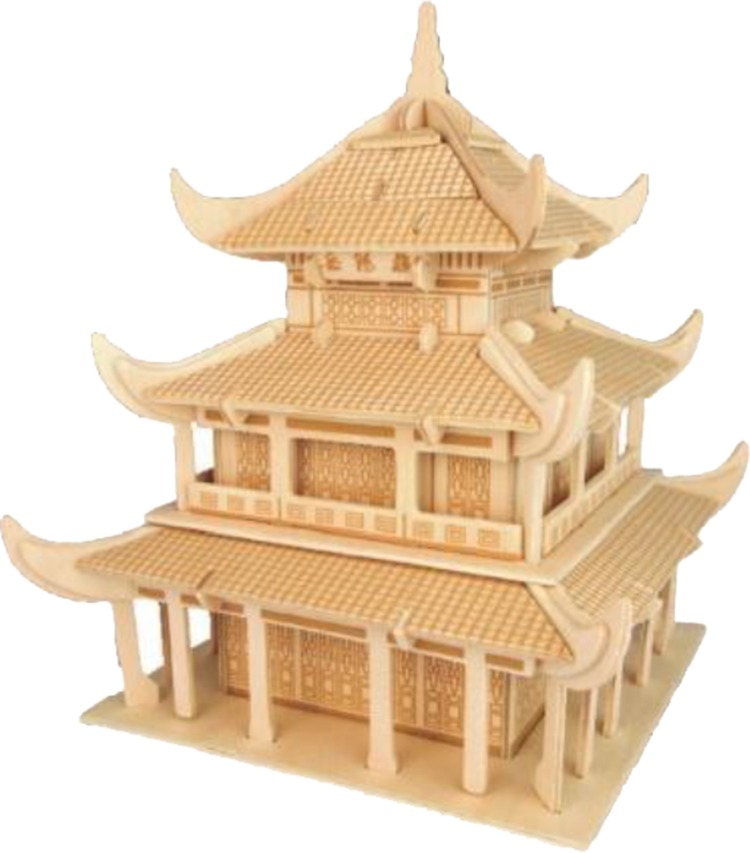
Reference materials used: *Yueyang Tower*.

A three-dimensional wooden model of *Yueyang Tower* was presented to children to construct (see **Figure 1**). *Yueyang Tower* is a famous ancient Chinese tower in Hunan province, China. The *Yueyang Tower* was chosen because the preschoolers had no prior block building experience with it, and the building features of the *Yueyang Tower* range from simple to complex. The model was made up of six wooden plates that were the size of 34 cm (length) × 21 cm (breadth) × 0.3 cm (thickness), was chosen. The model weighed 0.65 kg, and it was 19 cm (length) × 19 cm (breadth) × 23 cm (height).

#### Pictures of the Tower

For the second reference material, two colored pictures of the wooden model of *Yueyang Tower* (one front view, one side view) were printed on A4-sized sheet of paper.

### Measures

Block building performance was assessed using multiple measures. Using scores generated across these measures, three dependent variables were analyzed: block building skills, structure balance, and structural features. For detailed information about these three measures, see Appendix.

#### Block Building Skills

The measure of block building skills combined scores from two scales. First, constructions were rated based on the *Block Construction Scoring Scale* (Phelps and Hanline, 1999; Hanline et al., 2001, 2010): non-construction use of blocks (score of 0.5), linear constructions (scores ranging from 1 to 1.5), bidimensional/areal constructions (2–4.5), and tridimensional constructions (5–6.5). Block Construction Scoring Scale was intended to measure the complexity of block constructions, inter-rater reliability was between 0.83 and 1.00 (*M* = 0.95) when assessed across 65 children ranging in age from 16 to 75 months (Hanline et al., 2001). Constructions were also given a rating for tridimensional enclosure (7–9), from the *Block Building Measure* (Casey et al., 2008) with high inter-rater reliability (0.90–0.93). Thus, five classifications could be obtained using the measure of block building skills: Non-construction use of blocks, linear constructions, bidimensional/areal constructions, tridimensional constructions, and tridimensional enclosure.

#### Structural Balance

Based on a mixture of Study 1 and Study 2 as regards the *Measure of Structural Balance* and *Structural Balance Rating Scale* (Casey et al., 2012), inter-rater reliability was 0.91 and 0.87, respectively, six levels of rating consequently remained: stacking (rating of 1), bridging (rating of 2), bridging on a non-flat surface (rating of 3), scaffolding (rating of 4), balancing using counter-weights (rating of 5), and balancing using center- and counter-weights (rating of 6).

#### Structural Features

The measure of structural features developed for the present study based on the block building reference object *Yueyang Tower*. *Yueyang Tower* is a 3-story rectangular building completely of wood structure, including the bottom, the main tower, three layers of upturned eaves, and the roof. There are four classifications in the scale of structural features: bi-/tri-dimensional structure (scores of 1–1.5), basic structure (1–1.5), structural details (0.75–1.25), and representational play (1–1.5). A score was assigned to each classification independently, therefore, these four scores are summed to create a composite of total structural features.

### Procedure

#### Pre-test Preparation

Before the formal experiment, experimenters made classroom visits to observe block building performance across different grade levels and to negotiate with the preschool teachers in order to facilitate the forthcoming data collection for the experiment. The observation stage lasted 1 month. Three graduate students majoring in psychology received systematic training in order to become familiar with the data collection procedure.

#### Formal Test Procedure

Two rooms were used for assessment, a larger room for preschoolers in K2 and K3, and a smaller room for preschoolers in K1. Each room included unit blocks of varying shapes and sizes for the preschoolers to use. The experiment was conducted in either the morning or afternoon according to the respective schedules of the preschoolers across the three grade levels. Block activity was self-paced by the preschoolers. Half of the participants took wooden model of the tower as the reference material, the other half took pictures of the tower as the reference material.

During the course of the assessment, participants were given instructions that varied depending on social settings (work individually, work in a 5-member group, or work in a 10-member group). The instructions for the individual condition were as follows: “Let’s play block building games! You see there, it is a *Yueyang Tower* model for your reference, which requires you to build the *Yueyang Tower* using blocks. After you finish, you’ll receive a gift as a reward.” The only difference in instructions for the 5-group and 10-group building settings were the addition of the sentence “Please notice that each of you should build *Yueyang Tower* alone without the cooperation of others.”

### Scoring

For the purpose of offline scoring, children’s final block constructions were recorded with photographs taken from various angles, e.g., front, back, left, right, up, down and interior space. Three raters who were blind to the aims and hypotheses of the current study independently completed the scoring of those photographs of the 180 block constructions. Interrater reliability of the three measures, namely block building skills, structural balance, and structural features, was established using the Kendall coefficient of concordance among the three raters. Kendall’s *W* ranged from 0.952 to 0.992 (*M* = 0.974), *p* < 0.001, indicated high interrater reliability for the three measures. Five senior preschool teachers who taught block construction to preschoolers for 13 to 20 years rated the content validity of each measure using a 5-point Likert scale (1 = *low content validity*, 5 = *high content validity*). The mean rating was 4.89 ± 0.15, indicating the high content validity.

## Results

Outliers 3 *SD*s above or below the mean were trimmed during pre-processing of the data (e.g., Li et al., 2017a,b). A series of 2 (Gender: male, female) × 3 (Grade Level: K1, K2, K3) × 2 (Types of Model: wooden model, picture) × 3 (Social Settings: individual, 5-member group, 10-member group) multivariate analyses of variance (MANOVA) was carried out, for three dependent variables: block building skills, structural balance and structural features. The MANOVA analysis of gender, grade level, types of model and social settings yielded a Wilks’ Lambda = 0.926, *p* = 0.149; Wilks’ Lambda = 0.058, *p* = 0.000; Wilks’ Lambda = 0.949, *p* = 0.399 and 0.926, *p* = 0.000, respectively.

### Block Building Skills

There were significant main effects of gender, *F*(1,144) = 5.028, *p* = 0.026, η^2^ = 0.034, and grade level, *F*(2,144) = 159.670, *p* < 0.001, η^2^ = 0.689. Bonferroni *post hoc* pairwise comparison showed that, boys’ score for block building skills (*M* = 5.81, *SD* = 1.88) was significantly higher than that for girls (*M* = 5.48, *SD* = 1.84), *p* = 0.026. Scores in the K1 group (*M* = 3.50, *SD* = 1.75) were significantly lower than in K2 (*M* = 6.68, *SD* = 0.50), *p* = 0.000, and in K3 (*M* = 6.75, *SD* = 0.48), *p* = 0.000. There was neither a significant main effect of types of model, *F*(1,144) = 0.004, *p* = 0.950, nor of social settings, *F*(2,144) = 1.021, *p* = 0.363. However, these results were subsumed under a two-way interaction between types of model and social settings, *F*(2,144) = 3.049, *p* = 0.050, η^2^ = 0.041, and a three-way interaction between grade, types of model and social settings, *F*(4,144) = 8.322, *p* < 0.001, η^2^ = 0.188.

Analysis of the simple effects showed that when the K1 group was presented with a wooden model, building skills in the 10-group setting (*M* = 5.983, *SD* = 1.277) were significantly higher than in the individual setting (*M* = 5.522, *SD* = 1.974) or 5-group setting (*M* = 5.644, *SD* = 2.422), with no significant difference between the individual and 5-group setting. When the K1 group was presented with a picture as a model, building skills in the 5-group setting (*M* = 5.944, *SD* = 1.477) were significantly higher than in the individual setting (*M* = 5.574, *SD* = 1.904) and in the 10-group setting (*M* = 5.433, *SD* = 2.075), with no significant difference between the individual and 10-group setting.

### Structural Balance

There was a significant main effect of gender, *F*(1,144) = 6.675, *p* = 0.011, η^2^ = 0.044, and grade level, *F*(2,144) = 219.803, *p* < 0.001, η^2^ = 0.753, were both observed, with boys (*M* = 4.34, *SD* = 1.16) performing better than girls (*M* = 4.14, *SD* = 1.37), *p* = 0.011, and K2 (*M* = 5.00, *SD* = 0.000) and K3 (*M* = 5.00, *SD* = 0.000) performing better than K1(*M* = 2.71, *SD* = 1.16), *p* = 0.000. The interaction between gender and grade level was also significant, *F*(2,144) = 6.675, *p* = 0.002, η^2^ = 0.085. Specifically, no difference between boys and girls was observed in K2 and K3 (*M* = 5.000, *SD* = 0.000), but in K1, boys’ structural balance scores (*M* = 2.943, *SD* = 1.144) were significantly higher than girls’ scores (*M* = 2.495, *SD* = 1.154). There was neither a significant main effect of types of model, *F*(1,144) = 0.014, *p* = 0.906, nor of social settings, *F*(2,144) = 0.326, *p* = 0.722. However, these results are subsumed under a two-way interaction between types of model and social settings, *F*(2,144) = 5.157, *p* = 0.007, η^2^ = 0.067, and a three-way-interaction between types of model, social settings, and grade level, *F*(4,144) = 5.157, *p* = 0.001, η^2^ = 0.125.

Analysis of the simple effects showed that, for K1, but not K2 or K3, children who were given a wooden model showed worse performance in the individual setting (*M* = 2.311, *SD* = 1.123) compared to both the 10-group (*M* = 3.100, *SD* = 0.994) and 5-group (*M* = 2.800, *SD* = 0.837) settings. When the K1 children were given a picture as a model, building performance in the individual setting (*M* = 3.244, *SD* = 1.172) was significantly higher than in both the 5-group (*M* = 2.667, *SD* = 1.886) and 10-group (*M* = 2.100, *SD* = 0.738) settings.

### Structural Features

There was no main effect of gender, *F*(1,144) = 0.260, *p* = 0.611. There was a significant main effect of grade level, *F*(2,144) = 257.556, *p* < 0.001, η^2^ = 0.782, with K3 (*M* = 10.37, *SD* = 1.94) being significantly higher than K2 (*M* = 7.49, *SD* = 1.68), *p* = 0.000, and K2 being significantly higher than K1 (*M* = 3.10, *SD* = 1.46), *p* = 0.000. There was no main effect for types of model, *F*(1,144) = 0.844, *p* = 0.360. However, there was a significant main effect of social settings, *F*(2,144) = 3.165, *p* = 0.045, η^2^ = 0.042, with the best performance in the 5-member group, followed by the individual setting (*M* = 7.18, *SD* = 3.36) and then the 10-group setting (*M* = 6.56, *SD* = 3.34).

However, these main effects were subsumed under interaction effects. First, there was a two-way interaction between social settings and types of model, *F*(2,144) = 4.903, *p* = 0.009, η^2^ = 0.064. Analysis of the simple effects showed that, when given a wooden model, the structural features score in the individual setting (*M* = 7.28, *SD* = 3.68) was higher than in both the 5-group (*M* = 6.87, *SD* = 4.13) and 10-group (*M* = 7.26, *SD* = 3.25) settings. When given a picture as a model, the structural features score in the 5-group setting (*M* = 7.63, *SD* = 3.80) was higher than that of both the individual (*M* = 7.09, *SD* = 3.04) and 10-group (*M* = 5.87, *SD* = 3.33) settings.

## Discussion

The present study examined the impact of individual differences, types of model and social settings on three measures of block building performance (i.e., block building skills, structural balance and structural features). Performance varied depending on gender, grade level, and social settings, but not types of model used.

### Individual Differences

#### Gender Differences

Boys performed better than girls in block building skills and structural balance, consistent with studies showing that boys are significantly more likely than girls to engage in block building activities (Rubin, 1977), and they choose to play in the block area more often than girls (Snow et al., 2016). This finding contradicts other research suggesting a lack of gender differences (Moyer and Gilmer, 1956; Hanline et al., 2001). For instance, recent research showed that boys did not outperform girls on a measure of structural complexity, except that girls tended to build structures that included more symbolic features (Ramani et al., 2014). It should be noted that, unlike previous studies, we used multiple measures to assess block building, making it possible to detect gender differences on specific skills. Specifically, we found that boys performed better than girls in block building skills and structural balance. These skills have been reported to be associated with spatial development (e.g., Cohen and Emmons, 2017) and mathematic skills (e.g., Casey et al., 2012), and gender differences in these areas would be consistent with research showing that boys outperform girls in logical thinking and abstract awareness (Fennema et al., 1998). However, we found no significant gender differences in the other measures of block building, namely structure features. These skills are closely related to preschoolers’ spatial imagination, which might not be assessed well by the measures of block building used in this study. The fact that children in the current study were asked to copy a model rather than engage in free play might also have limited the chance to detect gender differences on these specific skills.

#### Grade Level Difference

Significant difference was found in block building scores depending on year in preschool. We found tridimensional constructions in K2 and K3 together with linear or bidimensional constructions in K1, consistent with other research (e.g., Reifel, 1984; Casey et al., 2008; Cohen and Emmons, 2017) showing developmental trends with respect to dimensionality in young children’s block building. Combining blocks in only one dimensional space appears to be the most common form of block play before 2 years old. Between the ages of 2 and 3, children begin to build in two dimensions. Between 3 and 4, they gradually build blocks in three dimensions. It is not until 4 and 5 years that children build multicomponent constructions, and show a considerable flexibility in block building. Thus, there is general agreement that changes in the spatial dimensionality emerge in an organized fashion and increase with age.

One factor that appears to influence the increase in block building skills is that older children spend more time with blocks than younger children (Clark et al., 1969), and the amount of time involved in block play has a positive effect on the complexity of block constructions (Halford et al., 1998; Hanline et al., 2001), including more spatial dimensions (Stiles-Davis, 1988; Stiles and Stern, 2001). Peer and teacher interactions in the block area also appear to promote block building performance (Trawick-Smith et al., 2016), and systematic teaching of block building skills accounts in part for block structure complexity (Casey et al., 2008).

### Types of Model

Vygotsky (1967, 1978) argued that play may be children’s chief means for developing and understanding symbols. Thus, block play may be a way for preschoolers to map the “signified” onto “signifier.” In the present study, we presented children with two types of model, namely a wooden model and pictures, and asked them to make a replicate. Interestingly, we found that the wooden model elicited more representational play than the picture, but children’s responses to the two types of representation did not differ in block building skills, structure balance, structure features.

Representational play refers to the representation of block constructions embedded with preschoolers’ detailed real-world experience, requiring imagination and demands (e.g., Norman and Bobrow, 1975; Duncan, 1980). Preschoolers’ processing difficulties are highly related to the detailed precisions of symbolic representation, i.e., the more detailed the symbolic representation, the easier it might be for preschoolers to process, which impact their performance of block building in turn. This perspective is consistent with Piaget’s model of cognitive development (Piaget, 1962a,b; Lourenço, 2016), in which preschoolers, typically in the preoperational stage, begin to engage in symbolic play and learn to manipulate symbols, but do not yet understand concrete logic. Thus, creating a three-dimensional structure based on a three-dimensional model (the model made of blocks) would be easier than making a three-dimensional structure based on a bidimensional model (the picture). When children were presented with a three-dimensional model they showed better representational play, but the dimensionality of the model did not affect other aspects of block building that might consume fewer cognitive resources.

### Social Settings

In the current study we measured block building performance in three social settings: building alone, in a group of 5 children, and in a group of 10 children. We found that block building performance was stronger when working in a small group than when working alone or working in a large group. This is consistent with early research showing that playing in pairs or small clusters elicited more intimate social interactions than were seen in larger clusters (Kinsman and Berk, 1979). The possibility that children would show better block building performance in smaller rather than larger groups is consistent with the “population interference effect”; that is, when members of a population are engaged in a cognitively demanding task, the efficiency of members’ performance is interfered with by mutual peer influence.

In the current study, the “population interference effect” was seen in the quality of structural features, rather than in the quality of the basic structure. Structure features refer to the degree of resemblance between the model or picture to be consulted and the children’s construction. Presumably, children in the 5-member groups encountered less mutual interference from peers than children in the 10-member group, allowing fuller expression of skills related to structural details. However, children in groups of 5 and in groups of 10 showed similar block building performance in terms of basic structure of *Yueyang Tower*, and both groups showed better performance than children working alone. Compared with other aspects of block building, the creation of a basic structure does not make as many cognitive demands, and so this skill may be less affected by interference from peers.

### Limitations and Future Research

The present study has several limitations that should be addressed in future research. First, the effects of symbolic representation results might have been extraneously influenced by the task difficulty in that both tasks, namely the model and the picture, are in visual modality that seems too easy to process among the participants. In this sense, will the effects of symbolic representation be relatively prominent with tasks of different modalities, e.g., verbal vs. visual? In future research a multimodal approach could be exploited to compare the verbal modality (e.g., the naming task; Cohen and Uhry, 2011) and visual modality (e.g., model or picture). Second, the samples that were only from one Chinese kindergarten might limit the generalization of our results. Future studies should use diverse samples from different areas of China (e.g., Hornung et al., 2017; Lo et al., 2017) and from other countries. Third, we used a cross-sectional design and longitudinal data will also be important to capture developmental change in the future studies.

## Conclusion

To the best of our knowledge, no previous study has used multiple measures to examine factors influencing block building among Chinese preschoolers. The present study makes the following contributions to the block building literature. First, it used multiple measures of block building in order to identify a range of specific skills. Second, it clarified the role of individual differences (gender, year in preschool) and methodology (types of model for children to copy, number of children at work) in predicting block building performance among Chinese preschoolers. Third, we were able to make some reference for both the scale development and the future research.

## Author Contributions

MT, RL, and ZM are responsible for research design, draft writing and editing. ZZ, WQ, and ZD are responsible for draft editing. RW, TY, and MJ are responsible for participants employment.

## Conflict of Interest Statement

The authors declare that the research was conducted in the absence of any commercial or financial relationships that could be construed as a potential conflict of interest.
