# Supplementary material for: The Impact of Individual Differences, Types of Model and Social Settings on Block Building Performance among Chinese Preschoolers
Source: Front Psychol. 2018 Jan 30;9:27. doi: 10.3389/fpsyg.2018.00027 (PMC5797599; doi:10.3389/fpsyg.2018.00027)
Supplement: Supplementary file 1 [file Presentation_1.pdf]

A Comprehensive Rating Scale of Preschool Children’s Block Construction Ability

Name________ Gender__________ Date of birth__________ Grade__________

Date_____ Observer_______ Is structure complete?_____ Final time__________

Height________ Number of blocks used_______ Types of blocks used_______

Level of block building skill________ Rating of structural balance________

Scoring for structural features_______

Subscale 1 Scale of Block Building Skills

Stage	Level	Description	Example	Rating score
No Constructions	0.5	Random block placement	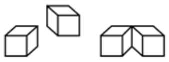	
Linear Constructions	1	Vertical linear arrangement	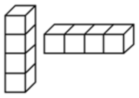	
	1.5	Horizontal linear arrangement		
Bidimensional Constructions	2	Horizontal/Vertical areal arrangement, no internal space, e.g., a wall, a floor	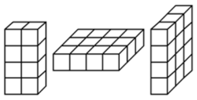	
	3	One block bridging upon two blocks with vertical internal space-arches	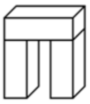	
	4	Block construction with horizontal internal space-enclosure with only 1 block high (no height)	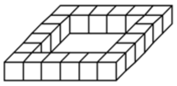	
	4.5	Regular/no gaps		
Tridimensional constructions	5	Bidimensional structure or horizontal internal space plus depth to make a tridimensional structure, e.g., 1 or more blocks placed in front or behind a arch structure	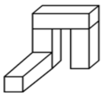	
	6	Tridimensional piles with no internal space	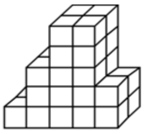	
	6.5	By using bridge and horizontal areal arrangement to make a tridimensional construction with internal space		
Tridimensional horizontal enclosure constructions	7	1 block-high enclosure (or partial enclosure)+layer of roof blocks-adds height to make a tridimensional structure. Irregular 1 block-high enclosure with roof-gaps/sloppy	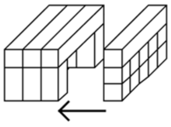	
	7.5	Regular(no gaps) 1 block-high enclosure with roof, or series of arches(tunnel + third wall-make a partial horizontal enclosure)		

	8	Irregular 2-block high enclosure-gaps/sloppy	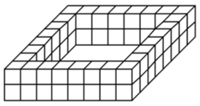	
	8.5	Regular (no gaps) 2-block high horizontal enclosure		
	9	2 blocks high + roof + divided internal space	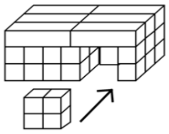	

Note: Adapted from Phelps & Handline, 1999; Hanline, Milton, & Phelps, 2001, 2010; Casey, Andrews, Schindler, Kersh, Samper & Copley, 2008

Subscale 2 Scale of Structure Balance

Rating	Example	Rating score
Rating of structural balance =1	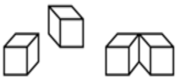	
Rating of structural balance =2	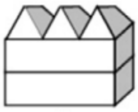	
Rating of structural balance =3	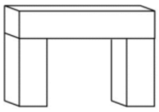	
Rating of structural balance =4	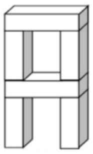	
Rating of structural balance =5	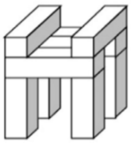	
Rating of structural balance =6	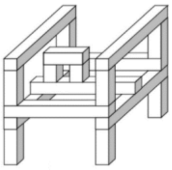	

Note: Adapted from Casey, Pezaris & Bassi, 2012

Subscale 3 Scale of Structural Features

Sub-levels	Scoring Criteria	Scoring	Rating score
Bi-/tridimensional structure	Simple linear/ bidimensional constructions	1	
	Relatively complex tridimensional constructions	1.5	
Basic structure	Refers to whether the preschool children could build the key elements of Yueyang Tower, including bottom, main building, upturned eaves, building top. Scored based on the basic structure. Note: If the construction presents more than three-layer structure, e.g., four-layer, scored as three-layer structure.		
	Present the bottom	1.25	
	Present one-layer main building	1	
	Present two-layer main building	1.25	
	Present three-layer main building	1.5	
	Present one-layer upturned eaves	1	
	Present two-layer upturned eaves	1.25	
	Present three-layer upturned eaves	1.5	
	Present the building top	1.25	
Structural details	Refers to whether the preschool children could present the reference object Yueyang Tower with the characteristics of symmetrical, well-ordered, and increasing progressively from top to bottom with layers, including 3 layers. Scored based on those structural details.		
	Present one-layer main building symmetrical and well-ordered	0.75	
	Present two-layer main building symmetrical and well-ordered	1	
	Present three-layer main building symmetrical and well-ordered	1.25	
	Present one-layer upturned eaves symmetrical and well-ordered	0.75	
	Present two-layer upturned eaves symmetrical and well-ordered	1	
	Present three-layer upturned eaves symmetrical and well-ordered	1.25	
	Present one increase progressively from top to bottom with layers	1.25	
	Present two increases progressively from top to bottom with layers	1.5	
	Present window, door, tiles, and railings	1.25	
Representational play	Representation of interior space, children's final structure includes internal structure or layout, e.g., chairs.	1.5	
	Representation of exterior space, building constructions with external structure or layout, e.g., steps.	1	

Note: Scale of Structural Features developed specially for the present study based on the reference object *Yueyang Tower*
